# Perception of an ambiguous figure is affected by own-age social biases

**DOI:** 10.1038/s41598-018-31129-7

**Published:** 2018-08-23

**Authors:** Michael E. R. Nicholls, Owen Churches, Tobias Loetscher

**Affiliations:** 1 School of Psychology, Flinders University, Adelaide, South Australia Australia; 2 School of Psychology, University of South Australia, Adelaide, South Australia Australia

## Abstract

Although the perception of faces depends on low-level neuronal processes, it is also affected by high-level social processes. Faces from a social in-group, such as people of a similar age, receive more in-depth processing and are processed holistically. To explore whether own-age biases affect subconscious face perception, we presented participants with the young/old lady ambiguous figure. Mechanical Turk was used to sample participants of varying ages from the USA. Results demonstrated that younger and older participants estimated the age of the image as younger and older, respectively. This own-age effect ties in with socio-cultural practices, which are less inclusive towards the elderly. Participants were not aware the study was related to ageing and the stimulus was shown briefly. The results therefore demonstrate that high-level social group processes have a subconscious effect on the early stages of face processing. A neural feedback model is used to explain this interaction.

## Introduction

Although face recognition is governed by low-level neural detection mechanisms^1^, it is also affected by seemingly incidental high-level social processes. A good example is the effect of social in- and out-groups. A social in-group is a collection of people with whom a person identifies as a member whereas an out-group is outside one’s identity. Social in- and out-groups can occur along multiple dimensions including race, sexual orientation, and age^2^. Hugenberg and Corneille^3^ demonstrated that social groups affect the processing of faces using the composite face paradigm. In this paradigm, participants are shown two faces that are the same at the top (eyes, forehead etc) but different at the bottom (mouth, chin etc). When asked to determine whether the top-halves are identical, participants erroneously indicate that the top halves are different. This effect is thought to reflect a holistic processing strategy where the bottom- and top-halves are processed as a composite. Composite processing can be disrupted by simply shifting the bottom- and top-halves so that they are misaligned – preventing holistic processing.

Hugenberg and Corneille^3^ used the composite face paradigm to examine holistic processing for faces that were either cast as an in-group (same university) or an out-group (different university). Same and different halves of faces were shown that were either aligned or misaligned from each of the groups. Results revealed a stronger composite face effect (i.e., holistic processing) for the in-group compared to the out-group and it was concluded that in-group faces are processed using a more holistic strategy. In addition to different styles of processing, Sporer^4^ suggests that group belonging also affects the depth of processing. In this model, in-group faces are thought to automatically receive in-depth processing and are categorised as individuals whereas out-group faces receive relatively limited processing resources and are not individuated.

The effect of in- and out-groups may be particularly relevant to the processing of faces of different ages. A common finding within the field is the own-age bias, where recognition memory is better for faces closer to one’s own age compared to other ages^5^. A meta-analysis by Rhodes and Anastasi^6^ revealed superior discrimination for own-age faces in both younger and older participants – though the advantage for own-age faces was weaker for older participants. The weaker effect for older participants is often explained by an exposure effect where older participants are exposed to a wider variety of ages compared to younger participants^7^. Rhodes and Anastasia^6^ suggest that these perceptual/learning effects should be integrated with social in- and out-group models to provide a comprehensive explanation of the own-age effect.

While a link between age-related social processes and facial recognition has been established, the level of consciousness at which this link occurs is less clear. The own-age bias for face recognition is classically demonstrated using a learning, retention and recognition paradigm^6^. In such tasks, participants are aware of the social categories and this knowledge may allow conscious high-level social processes to affect low-level face processing mechanisms (see: Ratner and Amodio^8^). The level of consciousness required for processing in- and out-group faces has been explored by Van Bavel, Packer and Cunningham^9^. They used fMRI to measure brain activity as participants made group-identity classifications for faces. On some trials, participants made explicit judgements related to group identity whereas judgements were made on an orthogonal dimension of race for other trials. Results revealed that the same neural centres were engaged irrespective of whether the group identity was overt or covert – suggesting an automatic, sub-conscious process.

Perceptual tasks, such as ambiguous figures, can also be used to investigate the level of conscious processing. Indeed, because reversals between different interpretations of a figure occur at different locations and levels of complexity within the visual processing system, they may be ideally suited to investigate the interplay between different levels of processing^10^. Balcetis and Dunning^11^ used ambiguous figures to investigate whether motivational states affect preconscious processing of visual stimuli. They assigned the two alternate perceptions of an ambiguous figure (whether a ‘I3’ is viewed as a ‘B’ or as ‘13’) with a positive or negative outcome and found that participants subconsciously perceived the version of the image that produced a positive outcome. Similarly, Van de Cruys, Schouten and Wagemans^12^ presented participants with ambiguous human light-point walkers, whose movement can be interpreted as either facing towards or away from the observer. They found that socially anxious participants were more likely to report the figure as facing away from them and therefore suggested that trait emotion can bias perception (also see Brugger & Brugger^13^).

**Figure 1:**
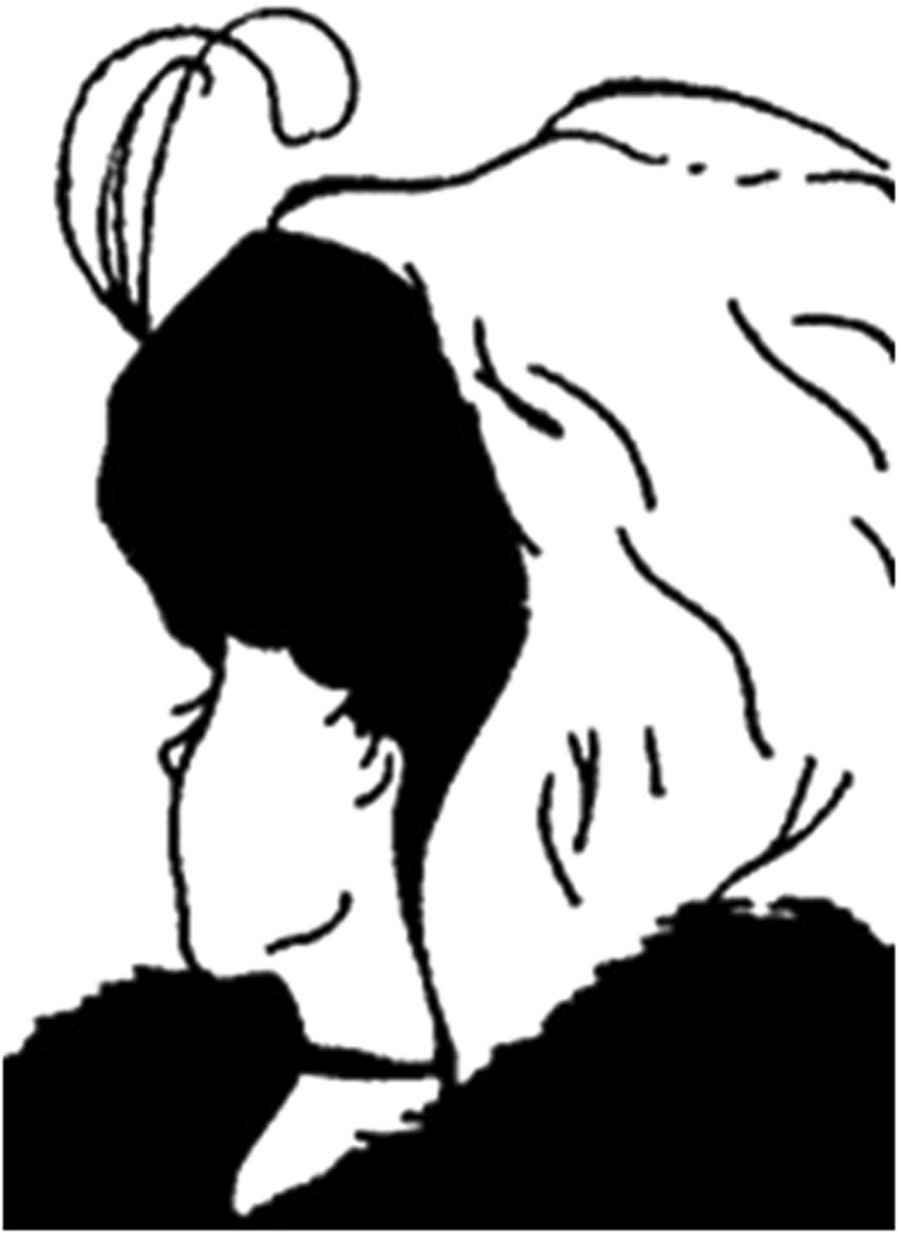
My Wife and My Mother-In-Law, by the cartoonist W. E. Hill, 1915. This media file is in the public domain in the United States. This applies to U.S. works where the copyright has expired, often because its first publication occurred prior to January 1, 1923.

Ambiguous figures provide an ideal means for exploring whether social/cognitive states can affect perception at a subconscious level. Given this utility, the current study used ambiguous stimuli to examine whether own-age social biases affect the basic sensory processing of faces. To do this, we used a well-known ambiguous image. The “my wife and my mother-in-law” illusion was introduced to the psychological literature by Boring^14^ and alternates between the perception of a young or an old woman (See Fig. 1). The young/old woman illusion was administered to a large sample of people of differing ages using Mechanical Turk. To ensure that conscious processes would have little impact on the perceptual task, the stimulus was only shown once to each participant for half a second. Participants were subsequently asked how old the woman was and it was predicted that participants would report their respective in-groups, with younger participants more predisposed to report a young woman and older participants more predisposed to report an old woman. It is likely, however, that the difference between younger and older respondents will not be bimodal. There is an overall bias to report the younger woman^15^ and it is also likely that older respondents will often report seeing a younger woman, in line with the weaker own-age effect for older participants reported by Rhodes and Anastasia^6^. It is therefore expected that the range of reported ages will be larger for older compared to younger respondents.

The initial sample from Mechanical Turk included participants from around the World, with the largest numbers coming from the USA and India, who are both prolific users of Mechanical Turk^16^. Initial checks of task compliance (see methods for details) revealed that a significant proportion of participants (55%) from India were not able to complete the task. The sample was therefore limited to respondents from the USA. The advantage of this sample is that it is relatively homogenous and also that Americans generally have more negative views toward ageing compared to Indians – especially in relation to socio-emotional processes^17^. In a study examining cultural differences in children’s attitudes towards the elderly, Zandi, Mirle and Jarvis^18^ wrote: “In the Western world, old age has often been conceived of as a period of life without meaningful roles” (p. 163). Bearing these socio-cultural practices in mind, we chose to use a sample from the USA, which would be most likely to yield an own-age effect.

## Method

### Participants

Mechanical Turk was used to sample 666 participants who were older than 18 years. Consistent with cultural analyses of Mechanical Turk^16^, the majority of users came from the USA (*n = *418) and India (*n = *225) with the remainder coming from 20 other countries (*n = *23). All participants were paid USD $0.30 for their time.

While Mechanical Turk has several distinct advantages for data collection^19^, there are also reports that users pay less attention to experimental materials^20^. To select attentive participants, we included two attention-check questions so that participants could be selected on an *a priori* basis (see procedure for details). Participants were also required to provide valid answers to the demographic questions as well as estimate the lady’s age to be older than 18 years.

Initial analyses of compliance revealed marked differences between the USA and India. For people from India, 55% failed the attention-check test whereas only 6% failed from the USA. Given the poor attention-check results for participants from India and the possibility that many of them may not have understood the task instructions, or that the young/old lady illusion is culturally specific, the current sample was limited to participants from the USA. There were therefore 393 participants (*m = *242, *f = *151) from the USA in the final sample. The mean age of the sample was 32.87 years (*SD = *10.07) with a range of 18 to 68 years. The distribution of age was positively skewed with a strong bias towards younger participants. This bias, which most likely reflects familiarity with computers, meant that only five participants were over 60 years of age. The method and experimental procedure of the present research was approved by, and carried out in accordance with, the guidelines of the Social and Behavioural Research Ethics Committee at Flinders University.

### Stimuli and Procedure

Participants were recruited using Mechanical Turk. Informed consent was obtained from all participants prior to participation. After agreeing to participate, demographic data were collected, including the participant’s age (in years), sex, and country of residence. Participants were then readied for the presentation of the young/old lady bistable image - copied from the one used by Boring^14^ (see Fig. 1). The ambiguous image was subsequently presented for 500 ms, after which the display was cleared. To verify that participants had seen the image in one of its forms, two questions were then asked: “Did you see a person or an animal?” (possible responses: person/animal/neither) and, if this was answered correctly, “What was the sex of the person?” (possible responses: male/female/don’t know). Participants who answered both questions correctly were then asked to estimate the age of the woman in years. The testing session was terminated for participants who gave incorrect responses to either of the attention-check questions. The entire testing session took less than five minutes.

## Results

**Figure 2:**
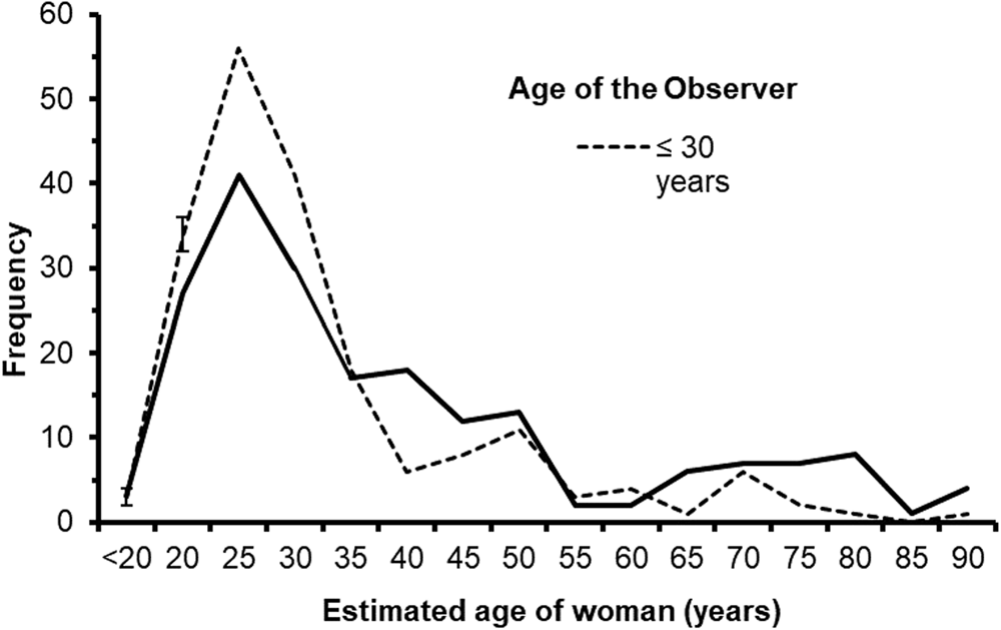
Graph shows mean estimated age of the ambiguous figure (in years) as a function of age of the observer and their country of residence. Error bars show the ± *SE* of the mean.

To gain an insight into how different age groups responded to the bistable image, we performed a median split of observer’s age and then divided the observers into a group of people who were 30 years or younger (*n = *195) or who were older than 30 years (*n = *198). The distributions of estimated age for the different groups is shown in Fig. 2. The first finding that is apparent from the figure is that the overall estimated age of the woman was skewed towards younger responses with a mean estimated age of 36.70 years (*SD = *16.65) and a mode of 25 years. Figure 2 also shows different profiles for the younger and older observers. The younger observers show an elevated frequency of age estimations around 25 years and relatively few estimations over 60 years. In contrast, older observers had a reduced peak at 25 years and made relatively more estimations over 60 years. The estimated age of the woman for the younger group was 33.51 (*SD = *13.55) years whereas the estimated age was 39.83 (*SD = *18.73) years for the older group. A *t*-test for independent samples revealed that the difference between the groups was highly significant [*t*(391) = 3.82, *p < *0.001, Cohen’s *d* = 0.386].

To obtain a stronger result and demonstrate that the difference between the younger and older groups was not related to a peculiarity of the median split, we also selected the very-youngest 10% of participants (aged between 18 and 22 years, *n* = 40) and the very-oldest 10% of participants (aged between 49 and 68 years, *n = *45). The estimated age for the very-young group was 33.85 (*SD* = 14.16) years whereas the estimated age for the very-old group was 45.91 (*SD* = 21.63) years. The estimated age difference of 12.06 years is larger than the difference observed for the median split (6.32 years) and the difference in estimated age for the very-young and -old groups was statistically significant [*t*(83) = 3.00, *p = *0.007, Cohen’s *d* = 0.659].

**Figure 3:**
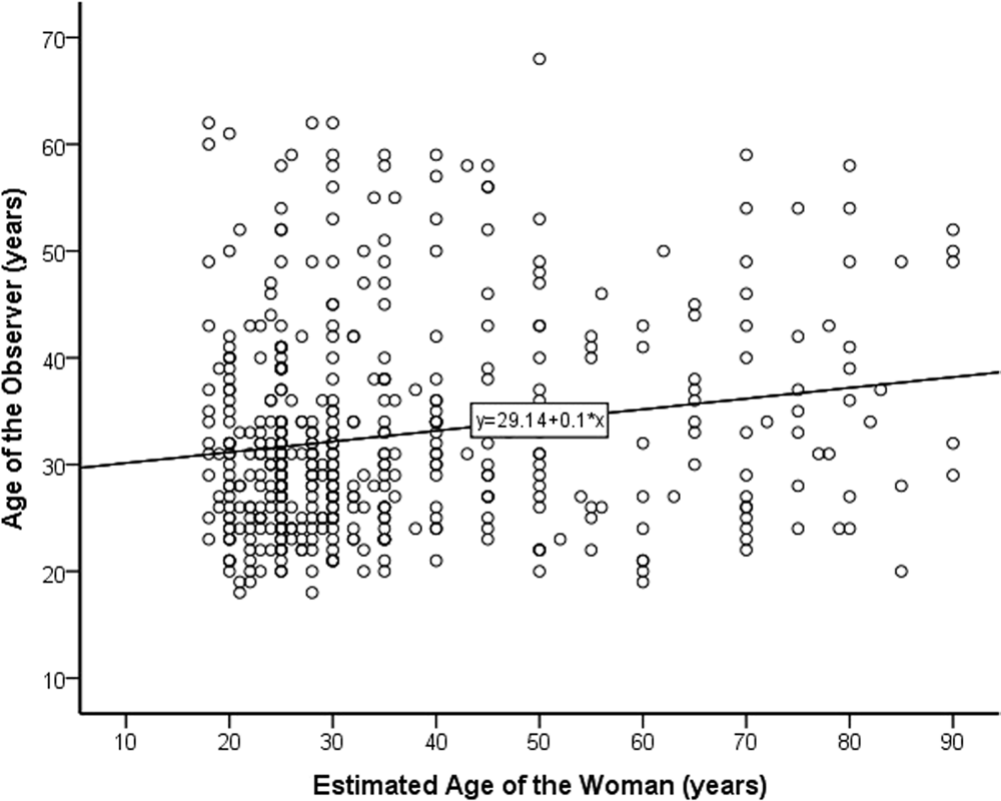
Association between estimated age of the woman in the figure and the age of the observer. The best-fitting linear regression is shown.

The relation between the observer’s age and the estimated age of the woman can also be investigated using a simple correlational analysis. Figure 3 shows a positive correlation between the observer’s age and the estimated age of the bistable illusion, which was statistically significant [*r*(393) = 0.237, *p < *0.001].

## Discussion

This study examined whether own-age biases affect the initial interpretation of an image at a subconscious level. To test this, the classic young/old lady ambiguous figure was administered to a group of participants of varying ages using Mechanical Turk. Although the estimated age data are bimodal, there is a bias towards reporting a younger woman. It is possible that this bias relates to a default ‘younger’ response. As noted by Georgiades and Harris^15^, participants are biased towards reporting a younger woman by 70%. This bias of response may be the default interpretation by the brain, which is only overcome when the social in-group favours an ‘older’ response.

A median split was used to sort the participants into groups of younger and older respondents. Analyses of the different groups revealed that younger participants estimated the woman’s age to be 6.3 years younger than the older participants. This difference in estimated age increased to 12.1 when the very-youngest and -oldest participants were selected. Both split analyses were supported by a simple correlational analysis, which showed that, as the age of the observer increased, so too did the estimated age of the woman. The consistency of the association between estimated age and participants’ age across the different types of split and the correlation analysis demonstrates that the effect is not an artefact of the way we analysed the data. The effect of the observer’s age on the estimated age of the woman is consistent with an own-age social group bias. Within the respective age-groups, participants have a bias towards processing faces of a similar age. A strong delineation between younger and older people in Western society in general and within the USA in particular^17,18^ may have precipitated social in- and out-groups, which is known to affect face processing.

The own-age bias may have been stronger for younger- compared to older-participants as reflected in lower standard deviations for the younger and very-young groups (*SD* = 13.55 & 14.16, respectively) compared to the older and very-old groups (*SD* = 18.73 & 21.63, respectively). A larger variation in estimated age for the older participants is in line with an exposure effect^7^ which may reduce the own age bias for this group^6^.

When participants engaged in the task, they were naïve in relation to the age-related aims of the study and did not expect the young/old ambiguous figure. The image was also displayed briefly for 500 ms. Both procedures ensured that any biases in the reported age of the woman reflected the operation of a preconscious perceptual process. Bearing this in mind, we believe that our data demonstrate that high-level social/group processes have a subconscious effect on low-level face detection mechanisms. Bar^21^ describes a neural mechanism to explain the effect of top-down facilitation of object recognition. In this model, a partially analysed version of the image is sent from early visual centres to the prefrontal cortex. This image then interacts with higher-level expectations of the image and is then sent as an ‘initial guess’ to the temporal cortex where it integrates with bottom-up mechanisms. In the current study, we believe that a partially analysed version of the ambiguous figure is passed through to frontal regions where social predispositions bias the interpretation towards an in-group outcome, which is subsequently fed-back to the decision-making mechanism.

Future research could rule out the possibility that the effect of the observer’s age on perceived age is specific to the bi-stable image used in this study. It is possible that participants simply estimate an age for the illusion that is closer to their own. This could be tested by simply picking a middle-aged face and asking participants to estimate the age of the face. Alternatively, the discrimination could be made orthogonal to the dimension of interest by asking participants to determine whether the face is looking to the side (old lady) or away (young lady) from the viewer.

## Data Availability

The datasets and syntax generated and analysed in the current study are available in the Open Science Framework repository, [https://osf.io/pk4sh].
